# Toxicokinetics of homosalate in humans after dermal application: applicability of oral-route data for exposure assessment by human biomonitoring

**DOI:** 10.1007/s00204-024-03704-7

**Published:** 2024-03-14

**Authors:** Katharina E. Ebert, Peter Griem, Tobias Weiss, Thomas Brüning, Heiko Hayen, Holger M. Koch, Daniel Bury

**Affiliations:** 1 Institute for Prevention and Occupational Medicine of the German Social Accident Insurance, Institute of the Ruhr University Bochum (IPA), Bürkle-de-la-Camp-Platz 1, 44789 Bochum, Germany; 2 Symrise AG, Mühlenfeldstrasse 1, 37603 Holzminden, Germany; 3 Institute of Inorganic and Analytical Chemistry, University of Münster, Corrensstrasse 48, 48149 Münster, Germany

**Keywords:** Homosalate, Ultraviolet filter, Sunscreen, Human biomonitoring, Toxicokinetics

## Abstract

Homosalate (HMS) is a UV filter used in sunscreens and personal care products as a mixture of *cis*- and *trans*-isomers. Systemic absorption after sunscreen use has been demonstrated in humans, and concerns have been raised about possible endocrine activity of HMS, making a general population exposure assessment desirable. In a previous study, it was shown that the oral bioavailability of *cis*-HMS (*c*HMS) is lower than that of *trans*-HMS (*t*HMS) by a factor of 10, calling for a separate evaluation of both isomers in exposure and risk assessment. The aim of the current study is the investigation of HMS toxicokinetics after dermal exposure. Four volunteers applied a commercial sunscreen containing 10% HMS to their whole body under regular-use conditions (18–40 mg HMS (kg bw)^−1^). Parent HMS isomers and hydroxylated and carboxylic acid metabolites were quantified using authentic standards and isotope dilution analysis. Further metabolites were investigated semi-quantitatively. Elimination was delayed and slower compared to the oral route, and terminal elimination half-times were around 24 h. After dermal exposure, the bioavailability of *c*HMS was a factor of 2 lower than that of *t*HMS. However, metabolite ratios in relation to the respective parent isomer were very similar to the oral route, supporting the applicability of the oral-route urinary excretion fractions for dermal-route exposure assessments. Exemplary calculations of intake doses showed margins of safety between 11 and 92 (depending on the approach) after single whole-body sunscreen application. Human biomonitoring can reliably quantify oral and dermal HMS exposures and support the monitoring of exposure reduction measures.

**Supplementary Information:**

The online version contains supplementary material available at 10.1007/s00204-024-03704-7.

## Introduction

Homosalate (HMS; 3,3,5-trimethylcyclohexyl salicylate (IUPAC); CAS registry no. 118–56-9) is a UV filter absorbing in the UVB region (280–315 nm). It is used worldwide (Shaath 2010) at maximum concentrations of up to 10% (EU, ASEAN) or 15% (USA, Australia) (ASEAN 2003; European Parliament and the Council 2009; Therapeutic Goods Administration 2021; U.S. Food & Drug Administration 2021) in sunscreens and personal care products such as creams, makeup, fragrances or lip care products (Danish Environmental Protection Agency 2015).

A recent market study on internationally available sunscreen products reported HMS in about 15% of investigated products, with similar usage frequencies in sunscreens meant for adults and for children (*n* = 444) (Jesus et al. 2022), while another study focusing on New Zealand reported HMS in 50% of products (*n* = 95) (Roh and Cheng 2022). Considering the widespread use of HMS, a release of HMS into the environment – e.g., via recreational activities such as swimming – appears likely, and indeed, HMS has been detected in a variety of environmental matrices, such as freshwater, seawater and wastewater, sediments, and aquatic organisms (Del Rosario et al. 2014; Bargar et al. 2015; Cuderman and Heath 2007; Cunha et al. 2018; He et al. 2019; Kameda et al. 2011; Mitchelmore et al. 2019; Nagtegaal et al. 1997; Sánchez Rodríguez et al. 2015). Furthermore, HMS has been detected in human urine (Ao et al. 2018) and breast milk samples (Liu et al. 2022; Schlumpf et al. 2010) of the general population.

Concerns have been raised regarding possible endocrine activity of HMS. While in vitro assays indicate possible estrogenic and anti-androgenic activity, no estrogenic activity was observed in either rat uterotrophic assays (European Chemicals Agency 2023; Schlumpf and Lichtensteiger 2001) or transgenic zebrafish assays (Schreurs et al. 2002). However, in 2021, the European Scientific Committee on Consumer Safety (SCCS) recommended a reduction of the permitted maximum concentration based on potential reproductive toxicity observed in an OECD 422 oral repeated dose study (Scientific Committee on Consumer Safety (SCCS) 2021). Consequently, an amendment of the European Cosmetics Directive, which will come into force in 2025, will restrict HMS to facial products only, excluding propellant spray products, with a maximum concentration of up to 7.34% (European Parliament and the Council 2022).

Given the toxicological and consumer relevance of HMS, quantitative assessments of human exposure are desirable. While dermal exposure is likely to be the major uptake route of HMS, additional oral HMS exposure via lip care products or hand-to-mouth contact can be expected. Hence, human biomonitoring is the tool of choice for exposure assessments, allowing a determination of the internal dose irrespective of the uptake route (Angerer et al. 2007). Based on comparison with other salicylates, it is likely that a considerable share of HMS will be metabolically hydrolyzed to salicylic acid (Belsito et al. 2007; Bury et al. 2019a). However, this metabolite is not specific for HMS. As ECHA has noted that different salicylates demonstrate different systemic toxicity in animal experiments (European Chemicals Agency 2018), a substance-specific biomonitoring approach via oxidized metabolites of the intact salicylic acid ester was considered essential.

The estimation of exposures via reverse dosimetry requires the prior elucidation of toxicokinetic data. Kinetic studies were previously performed in rats after both i.v. injection and topical application of HMS in gel (Kim et al. 2014) and their data have been used in physiologically based pharmacokinetic modeling approaches (Najjar et al. 2021). Furthermore, in humans, HMS plasma concentrations after sunscreen application under maximum-use conditions have been reported (Matta et al. 2020), thereby unequivocally showing systemic uptake of HMS. However, all of these studies treated HMS as a singular compound, while HMS is in fact a mixture of *cis*- and *trans*-isomers. We recently reported a rather constant relative *trans*-HMS (*t*HMS) share of 8–13% (and a respective *cis*-HMS (*c*HMS) share of 87–92%) in sunscreens from the German, US, and Canadian markets (*n* = 10) (Ebert et al. 2022), thereby contradicting older reports of HMS isomer mixtures with either 60% or 85% *t*HMS (Čajkovac 2000). Only for a labeled internal standard commercially available for HMS (2-hydroxybenzoic-*d*_4_ acid 3,3,5-trimethylcyclohexyl ester (Merck, prod. no. 49949)), we indeed observed a *c*HMS:*t*HMS ratio of 40:60, confirming the existence of HMS with starkly differing *cis:trans* ratios. While studies performed by Symrise used HMS containing around 87% *c*HMS (personal communication, unpublished), the *cis:trans* ratio of HMS in other past toxicity or dosing studies is unknown and might have ranged from a relative share of 8 to 85% of *t*HMS. It is well-established that stereoisomers may show differences in both pharmacodynamics and pharmacokinetics (Berners-Price and Kuchel 1990; Cleare and Hoeschele 1973; Chan et al. 1972; Ding et al. 1997), and indeed, we showed in a recently published oral metabolism study (Ebert et al. 2022) that the oral bioavailability of *c*HMS was tenfold lower than that of *t*HMS, and that the excretion of *c*HMS-derived specific, oxidative metabolites was even two orders of magnitude lower. These major differences in toxicokinetics between *c*HMS and *t*HMS indicate that data which fail to differentiate between these two isomers may be ill-suited for the use in exposure and risk/safety assessments.

Dermal absorption is known to be dependent on a variety of factors such as skin thickness, exposure duration, or formulation of the vehicle (European Centre for Ecotoxicology and Toxicology of Chemicals (ECETOC) 1993). This precludes the derivation of generally applicable dose recoveries, which would be useful for toxicokinetic modeling or reverse dosimetry. In contrast, oral metabolism studies bear the advantage of allowing a precise determination of the administered dose and, consequently, the reliable determination of relative dose recoveries. Nevertheless, the applicability of the previously obtained oral HMS data (i.e., in particular the urinary excretion fractions (F_ue_)) for exposure assessments should be demonstrated in a dermal exposure scenario. Accordingly, this study aimed to investigate the toxicokinetics of *c*HMS, *t*HMS, and specific oxidative HMS metabolites after dermal HMS exposure in a sunscreen application study under regular-use conditions. Analysis and data evaluation were performed analogously to the previous oral study (Ebert et al. 2022). Systematic comparison of data for the oral and dermal uptake routes allowed us to demonstrate a straightforward approach for a holistic HMS exposure assessment, which accurately describes multi-route exposures as oral-dose-equivalent intakes.

## Materials and methods

### Chemicals and reagents

**Fig. 1:**
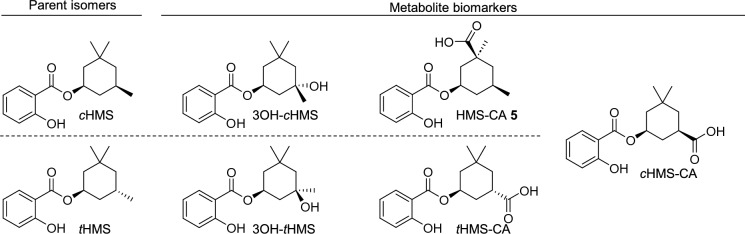
Chemical structures of the parent HMS isomers and their respective quantitatively investigated HMS metabolite biomarkers (only one enantiomer shown for simplicity). *c*HMS-CA was shown to be formed from both *c*HMS and *t*HMS

A commercial sunscreen (Coppertone Sport Sunscreen Lotion SPF 50) was purchased online and used for the dermal application study. Commercial HMS for use as analytical standard was obtained as Neo Heliopan® HMS (Symrise AG, Holzminden, Germany; 99.8%) and diastereomerically pure *t*HMS was purchased from TLC Pharmaceutical Standards Ltd. (Newmarket, ON, Canada) (H-045003, 97.3%). Aryl-deuterated HMS (2-hydroxybenzoic-*d*_4_ acid 3,3,5-trimethylcyclohexyl ester; HMS-*d*_4_, mixture of isomers) was purchased from Merck (49949,  ≥ 98.0%) for use as an internal standard. HMS metabolite analytical standards and aryl-deuterated internal standards (*t*HMS-CA,* t*HMS-CA-*d*_4_, *c*HMS-CA, *c*HMS-CA-*d*_4_, HMS-CA **5**, 3OH-*t*HMS, and 3OH-*c*HMS) were obtained by custom synthesis in cooperation with the Max Planck Institute for Multidisciplinary Sciences, Göttingen, Germany. See Ebert et al. (2021) and (2023) for further reagents, including a detailed description of the syntheses of the metabolite standards. See Fig. 1 for the structures of the non-labeled analytes.

### Determination of HMS isomer ratios

HMS content and the *cis:trans*-HMS isomer ratio of the obtained sunscreen product were determined using LC–UV as described in Ebert et al. (2022). See Section S-1 of the supplementary information for more detailed information.

### Study design

Dermal sunscreen application took place in late February to early April 2022. Volunteers were asked to abstain from the use of HMS-containing products in the time frame between 2 weeks before and 96 h after sunscreen application. Four healthy Caucasian volunteers (two male, two female; age: 29–49 years, 65–101 kg body weight (bw)) were recruited, three of whom had previously also participated in the oral study described in Ebert et al. (2022). The insides of the elbows were taped off to prevent contamination during blood sample collection. Each volunteer was asked to apply sunscreen in a regular-use scenario (i.e., using the amount of sunscreen they would normally use) to at least 80% of their body, estimated according to the rule of nines (arms, legs, trunk and neck; excluding feet, area covered by underwear, and part of the head). The volunteers washed their hands thoroughly and, once the sunscreen had dried, dressed in loose and short clothing (sleeveless shirts or T-shirts, short trousers). The skin was not occluded. The area surrounding the nose and mouth was excluded to prevent accidental oral or mucous membrane exposure and volunteers avoided hand-to-face contact until they took a shower approximately 10 h after application. The sunscreen bottle was weighed before and after application to calculate the amount of applied sunscreen. Estimated sunscreen application for each of the volunteers was 1.0, 1.4, 1.9, and 0.9 mg cm^−2^, respectively (using the Mosteller formula (Mosteller 1987; Vu 2002) for estimation of body surface area). Blood samples (5 mL) were collected in EDTA tubes (K3E, Sarstedt) before (t_0_) and 1, 2, 3, 4, 6, 8, 24, 32, and 48 h post-application and immediately centrifuged at 1200 *g* for 10 min to isolate the plasma. Urine samples were collected completely and consecutively in 250 mL or 500 mL polyethylene containers for 96 h after application, including one sample taken shortly before sunscreen application (t_0_). The collection times were recorded by the volunteers and sample volumes were determined gravimetrically (assuming a simplified urine density of 1 g mL^−1^). Plasma and urine samples were stored at − 20 °C until analysis. Urinary creatinine content was determined by contract analysis (L.u.P. GmbH Labor und Praxisservice, Bochum, Germany) based on the principle described by Jaffe (Jaffe 1886).

### Quantification of metabolites

The obtained plasma and urine samples were analyzed for HMS and its specific oxidative metabolites as previously described for the oral study in Ebert et al. (2022) using an online-SPE-LC–MS/MS method. In total, two parent isomers, five carboxylic acid metabolite isomers, and eleven alkyl-hydroxy metabolite isomers (of which two peaks co-eluted) were investigated quantitatively or semi-quantitatively, and a further four aryl-hydroxylated metabolites were analyzed qualitatively. At the time of the study, authentic analytical standards were available for *c*HMS, *t*HMS, 3OH-*c*HMS, 3OH-*t*HMS, *c*HMS-CA and *t*HMS-CA (Fig. 1). Where no authentic analytical standard was available for calibration, the calibration function of the closest-eluting available constitutional isomer was used for surrogate calibration. During method validation, 3OH-*t*HMS and 3OH-*c*HMS showed better accuracies when using *c*HMS-CA-*d*_4_ as an internal standard compared to *t*HMS-CA-*d*_4_, and *c*HMS-CA-*d*_4_ was therefore used as surrogate internal standard. For the remaining HMS metabolites without an authentic stable isotope-labeled internal standard, the closest-eluting available HMS-CA-*d*_4_ isomer was used as surrogate, i.e., *t*HMS-CA-*d*_4_ for HMS-CA **2** and *c*HMS-CA-*d*_4_ for the rest. See Fig. 3 for the nomenclature of the metabolites and Table S2 in the supplementary information for a detailed overview of the different metabolite isomers, retention times, and calibration information.

### Confirmation of HMS-CA 5 relative dose recovery

An analytical standard for the *c*HMS-specific metabolite HMS-CA **5** (Fig. 1) could only be obtained after the analysis and evaluation of the dermal study were already concluded (see Ebert et al. (2023) for the standard synthesis as well as the description and revalidation results of the analytical method). To enable as accurate a comparison as possible of HMS-CA **5** elimination between the oral and dermal pathways and provide the most accurate F_ue_s for future application in HMS exposure assessments, the revalidated method was used to analyze pooled urine samples of both the dermal (96 h) and the previously described oral (48 h) study (Ebert et al. 2022), and the respective dose recoveries were calculated for each volunteer after dermal and oral HMS exposure. The comparability of the two methods was shown by also analyzing the metabolism samples of one volunteer using the method described in Ebert et al. (2023) and comparing the two excretion curves (see Fig. S2).

### Statistical analysis

Analyst 1.7.3 and MultiQuant 3.0.3 (Sciex, Darmstadt, Germany) were used for instrument control and quantitative data analysis of LC–MS/MS measurements. Further data analysis was performed using Microsoft Excel 2019 (Microsoft Corporation, Redmond, U.S.A). Terminal half-times were calculated using urine samples ≥ 36-h post-application and the equation *t*_1__/2_ = ln(2)/*k* (Byers and Sarver 2009). *k* is the kinetic constant of the exponential decline in the excretion rate (*ER* in µg h^−1^), described by the equation *ER*(*t*) = *ER*_max_ × exp(-*kt*), and was obtained by exponential regression of *ER* vs. the midpoint of each time interval (*t* in h). The calculation of background-corrected concentrations, relative dose recoveries, and relative metabolite excretions is described in Section S-3 of the supplementary information. Figure 1 was created using ChemDraw 22 (PerkinElmer Informatics, Waltham, MA, USA) and Microsoft Powerpoint 2019. Figure 5 was created using OriginPro Version 2021b (OriginLab Corporation, Northampton, MA, USA) and Microsoft Powerpoint 2019. All other figures were created using Microsoft Excel and Powerpoint 2019.

## Results and discussion

### HMS content and isomer ratios of the sunscreen product

We previously demonstrated marked differences in bioavailability and metabolism between *c*HMS and *t*HMS (Ebert et al. 2022). Consequently, the knowledge of HMS isomer ratios was also essential for the investigation of HMS metabolism after dermal exposure. Therefore, it was necessary to determine the HMS isomer ratios in the applied sunscreen product. In this process, we also verified its declared total HMS content of 10% (see Section S-1 of the supplementary information). The relative share of 12.6% *t*HMS in total HMS was in agreement with a recent analysis of ten different sunscreens and personal care products (Ebert et al. 2022), in which *t*HMS shares of 8–13% were observed.

### Toxicokinetics of HMS and its specific oxidative metabolites after dermal administration

#### Toxicokinetics in plasma

**Fig. 2:**
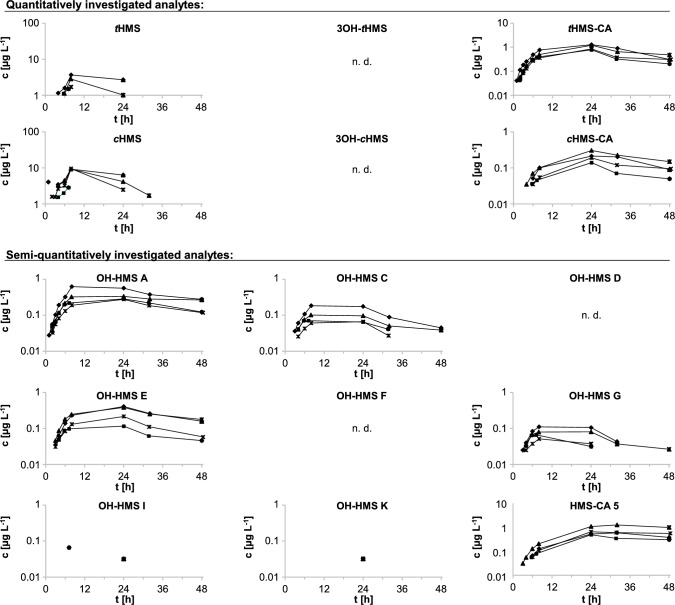
Plasma concentration–time profiles of HMS and its metabolites (total concentrations after deglucuronidation) after dermal sunscreen application (10% HMS, *cis:trans* 87.4:12.6). The four different data markers represent the four volunteers. In the volunteer represented by square markers, the 8-h sample had to be collected as early as 7.2 h post-dose due to time constraints. HMS-CA **2**, HMS-CA **3**, 3OH-*t*HMS, 3OH-*c*HMS and OH-HMS **D** and** F** concentrations were below the LOQ in all samples

Key toxicokinetic data (c_max_, t_max_) are listed in Table 1. Semi-logarithmic plasma concentration–time profiles of parent HMS and the HMS metabolites are shown in Fig. 2.

**Table 1:** Toxicokinetic parameters^a^ of the quantitatively and semi-quantitatively investigated metabolites and the two parent isomers in plasma after dermal sunscreen application (10% HMS, *cis:trans* 87.4:12.6)

	c_max_ [µg L^−1^]^b^	t_max_ [h]^b^
*t*HMS	2.4 (1.5–3.7)	7.2–8
*c*HMS	7.7 (2.8–9.5)	7.2–8
*t*HMS-CA (HMS-CA **1**)	1.0 (0.78–1.3)	24
*c*HMS-CA (HMS-CA **4**)	0.21 (0.14–0.31)	24
*HMS-CA ****5***^*d*^	0.74 (0.49–1.2)	24–32
3OH-*t*HMS (OH-HMS **B**)	< LOQ	n. a.^c^
3OH-*c*HMS (OH-HMS **H**)	< LOQ	n. a.^c^
*OH-HMS ****A***	0.38 (0.28–0.62)	8–24
*OH-HMS ****C***	0.11 (0.066–0.18)	7.2–24
*OH-HMS ****D***	< LOQ	n. a.^c^
*OH-HMS ****E (E***_***1***_** + *E***_***2***_***)***	0.28 (0.12–0.41)	24
*OH-HMS ****F***	< LOQ	n. a.^c^
*OH-HMS ****G***	0.077 (0.052–0.11)	7.2–24
*OH-HMS ****I***	0.024 (0.032–0.067, n = 2)	7.2–24
*OH-HMS ****K***	0.033 (n = 1)	24
*HMS-CA ****2***	< LOQ	n. a.^c^
*HMS-CA ****3***	< LOQ	n. a.^c^

Ranges are given in parentheses. Semi-quantitatively analyzed metabolites are given in italics. Terminal plasma half-times could not be determined due to too few samples above the LOQ

^a^c_max_: peak concentration; t_max_: time of peak concentration

^b^true maxima likely to be between 8- and 24-h post-application; maxima could not be accurately determined due to lack of samples in this time period

^c^could not be determined due to no samples > LOQ

^d^semi-quantitative determination using *c*HMS-CA calibration function

All plasma pre-dose concentrations were below the LOQ (1, 1.5, and 0.025 µg L^−1^ for *t*HMS, *c*HMS, and the oxidative metabolites, respectively). Similar to oral exposure, the parent HMS isomers exhibited the highest plasma concentrations (2.4 µg L^−1^ *t*HMS and 7.7 µg L^−1^ *c*HMS), followed by the carboxylic acid metabolites *t*HMS-CA (1.0 µg L^−1^) and the *c*HMS-specific carboxylic acid metabolite HMS-CA **5** (0.75 µg L^−1^). The hydroxylated metabolites OH-HMS **A** and OH-HMS **E** followed at 0.38 µg L^−1^ and 0.28 µg L^−1^, respectively. Concentrations of 3OH-*t*HMS, 3OH-*c*HMS, OH-HMS isomers **D** and **F**, and HMS-CA isomers **2** and **3** were below the LOQ in all samples.

Maximum plasma concentrations of the parent isomers and all metabolites were reached markedly later than after oral HMS exposure (1–2 h post-dose (Ebert et al. 2022)). It is known that the skin may act as a reservoir (Kemppainen et al. 1991; Chu et al. 1996), resulting in delayed and continued systemic absorption even after the end of the dermal application period. In the case of parent HMS, maximum concentrations of both isomers were observed in the 8-h sample; however, due to the lack of plasma samples between 8 and 24 h, a more precise allocation of the true maximum is not possible.

After topical application of HMS in rats, maximum plasma concentrations were reached after 12 h (Kim et al. 2014). In humans, the kinetics of HMS in plasma after single sunscreen application was recently investigated by Matta et al. (2020), and relatively constant plasma concentrations were observed between 8 h post-dose and the end of the sampling period (23 h post-dose, subjects did not shower during this period). Neither study provided information on HMS isomers; nevertheless, both studies agree with our observations that maximum concentrations occur at 8 h post-dose or later.

In the case of the major HMS metabolites (*t*HMS-CA, OH-HMS **A**, OH-HMS **G**), with the exception of HMS-CA **5**, maximum concentrations were generally observed later than the parent isomers (in the 8- or 24-h samples). The concentration–time curves indicate that the true maxima lie somewhere in between 8 and 24 h. In the case of HMS-CA **5**, the *c*HMS-specific carboxylic acid metabolite, plasma concentrations increased only slowly until 24 h post-dose, with c_max_ either in the 24-h or 32-h plasma sample (depending on the volunteer), and remained close to these concentrations also in the 48-h post-dose plasma sample. These findings are similar to those obtained after oral HMS exposure (Ebert et al. 2022), where also no major decrease in HMS-CA **5** concentrations was observed between t_max_ and the last data point (24 h post-dose).

#### Urinary elimination kinetics

**Fig. 3:**
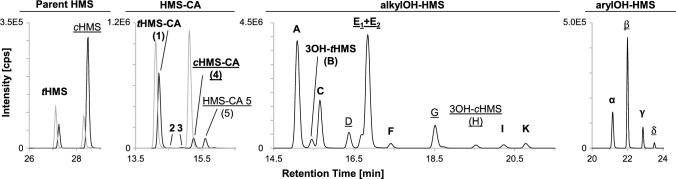
Exemplary chromatogram in one urine sample 5 h after dermal sunscreen application (10% HMS, relative *t*HMS content 12.6%). In addition to the two parent isomers, five carboxylic acid (HMS-CA **1**–**5**) and eleven alkyl-hydroxylated (OH-HMS **A**–**K**, with OH-HMS **E** consisting of at least two co-eluting peaks) and four aryl-hydroxylated metabolites (arylOH-HMS **α**–**δ**) were observed. The names of *t*HMS-derived metabolites are shown in bold and those of *c*HMS-derived metabolites are underlined (*c*HMS-CA is formed from both *t*HMS and *c*HMS and for the co-eluting alkyl-OH-HMS isomers **E**_**1**_ and **E**_**2**_, one is most likely formed from *c*HMS and one from *t*HMS (Ebert et al. 2022))

In total, the four volunteers provided 31, 35, 47, and 37 urine samples with total volumes of 6370, 7175, 10123, and 6506 mL, including the t_0_ samples. Pre-dose concentrations of the investigated metabolites were below the LOQ with the exception of *t*HMS-CA and the OH-HMS isomers **C** and **G** in two volunteers, and OH-HMS isomers **A** and **E** in three volunteers. However, their concentrations were rather low, more than two orders of magnitude below the respective maximum concentrations (creatinine-corrected values, compared separately for each metabolite and volunteer). All quantitatively investigated biomarkers (*t*HMS, *c*HMS, *t*HMS-CA, *c*HMS-CA, 3OH-*t*HMS and 3OH-*c*HMS) and most of the 12 semi-quantitatively investigated biomarkers became quantifiable in the post-application urine samples and remained so until the end of the sampling period 96 h post-application. See Fig. 3 for an exemplary post-dose chromatogram and metabolite isomer peak nomenclature.

**Fig. 4:**
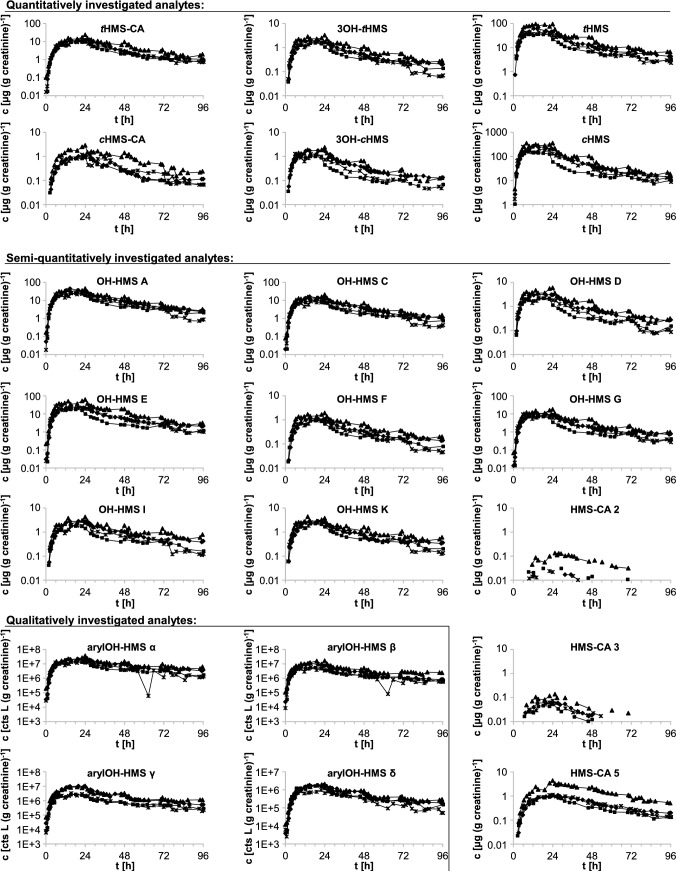
Urinary elimination kinetics of HMS and its metabolites after dermal sunscreen application. Data are shown for all four volunteers after dermal application of 12.6–26.1 g sunscreen, resulting in doses of approximately 18–40 mg HMS (kg bw)^−1^ (*cis:trans* isomer ratio of 87.4:12.6). The four different data markers represent the four volunteers. The semi-logarithmic plots show the creatinine-adjusted concentrations in [µg (g creatinine)^−1^] or creatinine-adjusted peak areas, each vs. the time of sample collection. See Fig. S3 for unadjusted concentrations and excretion rate–time curves of the quantitatively determined metabolites and HMS-CA **5** and Fig. S4 for the creatinine-adjusted concentrations normalized to the HMS dose

Creatinine-adjusted concentration–time curves or peak area–time curves (in the case of the qualitatively analyzed arylOH-HMS isomers) are shown in Fig. 4. Creatinine adjustment was used to compensate for fluctuating urine dilution and resulted in considerably smoother elimination curves. See Fig. S3 for the unadjusted concentration–time and excretion rate–time curves of the quantitatively analyzed metabolites as well as HMS-CA **5**. Despite the dermally applied doses differing by a factor of about 2 (18–40 mg HMS (kg bw)^−1^), the elimination kinetics were very similar for all four volunteers. Normalization to the applied dose (in g HMS · (kg bw)^−1^) further improved the agreement for the creatinine-adjusted concentration–time curves (see Fig. S4).

Maximum concentrations c_max_ (unadjusted and creatinine-adjusted), times of maximum creatinine-adjusted concentrations *t*_max_, and terminal half-times are summarized in Table 2. Maximum creatinine-adjusted concentrations reached a plateau around 6–24 h post-application for most metabolites and across all four volunteers. In a previous dermal pilot study in one volunteer (Bury et al. 2019a; Ebert et al. 2021), maximum creatinine-adjusted concentrations for metabolites of both HMS and the structurally related 2-ethylhexyl salicylate (EHS) were reached after 9 h, which is within the range observed herein. Due to the observed concentration plateau and a previously shown influence of the product formulation on the skin permeation of HMS (Kim et al. 2014), these values should be treated with caution, and hence only ranges but no mean values are listed in Table 2. Nevertheless, maximum concentrations clearly occur later than after oral exposure (2–7 h).

**Table 2:** Urinary elimination kinetics^a^ of quantitatively and semi-quantitatively investigated metabolites and parent HMS in four volunteers after dermal sunscreen application (10% HMS, *cis:trans* 87.4:12.6)

	c_max_ [µg L^−1^]	c_max_ [µg (g creatinine)^−1^]	t_max_ [h]	t_1/2_
*t*HMS	102 (75–121)	62 (36–96)	6–15	29 (24–35)
*c*HMS	420 (320–538)	261 (158–361)	6–24	27 (22–32)
*t*HMS-CA (HMS-CA **1**)	22 (20–23)	14 (9.6–24)	22–24	24 (21–29)
*c*HMS-CA (HMS-CA **4**)	2.4 (2.0–2.9)	1.5 (0.93–2.9)	22–24	21 (18–28)
*HMS-CA ****5***	2.8 (2.1–4.0)^c^	1.9 (1.0–4.4)^c^	24–27	24 (22–29)
3OH-*t*HMS (OH-HMS **B**)	3.5 (3.0–4.0)	2.2 (1.6–3.3)	15–24	26 (17–36)
3OH-*c*HMS (OH-HMS **H**)	2.6 (2.1–3.5)	1.6 (1.0–2.4)	6–24	28 (19–35)
*HMS-CA ****2***^*b*^	0.069 (0.027–0.12)	0.048 (0.017–0.11)	22–35	n. a.^d^
*HMS-CA ****3***^*b*^	0.12 (0.12–0.14)	0.082 (0.053–0.14)	24–25	n. a.^d^
*OH-HMS ****A***	58 (41–90)	35 (25–45)	15–24	23 (15–30)
*OH-HMS ****C***	22 (18–33)	14 (8.9–21)	15–24	23 (17–28)
*OH-HMS ****D***	5.8 (4.5–6.6)	3.5 (2.2–5.9)	6–24	24 (18–33)
*OH-HMS ****E**** (****E***_***1***_ + ***E***_***2***_*)*	52 (38–61)	33 (18–63)	6–24	25 (18–33)
*OH-HMS ****F***	2.1 (2.0–2.4)	1.3 (0.95–2.0)	15–24	27 (19–41)
*OH-HMS ****G***	19 (15–21)	11 (7.1–18)	6–24	27 (20–41)
*OH-HMS ****I***	4.2 (3.5–5.6)	2.8 (1.8–4.2)	18–24	33 (18–43)
*OH-HMS ****K***	5.0 (3.8–5.9)	3.0 (2.3–4.4)	14–20	31 (18–46)

Data are shown as mean values with ranges in parentheses, except for t_max_, for which only ranges are shown. Semi-quantitatively analyzed metabolites in italics

^a^c_max_: unadjusted and creatinine-adjusted peak concentrations, t_max_: time of creatinine-adjusted peak concentration, t_1/2_: terminal half-time, determined from excretion rate–time curves ≥ 36 h post-dose

^b^data for HMS-CA isomers **2** and **3** are less reliable due to overlap with *t*HMS-CA tailing, low concentrations, and a minor matrix peak co-eluting with HMS-CA **3**

^c^semi-quantitatively investigated using *c*HMS-CA calibration function

^d^could not be determined due to too few samples above the LOQ

Similarly, and as expected for the dermal uptake route, excretion was slower than after oral exposure, with only 30–61% of the recovered metabolites and parent HMS being excreted within the first 24 h (mean values across the four volunteers, assuming 100% excretion after 96 h and using background-corrected data; see Table S3 for detailed data), compared to > 70% after oral dose. Excretion was noticeably slower for HMS-CA **5**, with 30% excretion after 24 h compared to 41–61% for the other metabolites, which is in agreement with the observed slower plasma elimination.

With the exception of HMS-CA **2** and **3**, as well as 3OH-*c*HMS in one volunteer, all metabolites were still quantifiable in urine 96 h after the single application, showing that the investigated biomarkers are sufficiently sensitive to assess HMS exposure for 4 days after a single sunscreen application. Given the long terminal half-times in urine of around 24 h for both parent HMS and the metabolites, it is expected that repeated sunscreen application will lead to accumulation of HMS and thus higher analyte concentrations, which is supported by observations made by Matta et al. (2020) for parent HMS.

#### Glucuronidation of HMS metabolites and parent HMS

The glucuronidation status was investigated in 8- and 24-h plasma samples and 0–24-h pooled urine samples. The results are listed in Table S4. In urine, the majority of parent HMS and their metabolites (> 88%) were excreted as glucuronides, which is in agreement with the observations made after oral HMS exposure (parent HMS isomers ≥ 93%, 3OH-*c*/*t*HMS and *c*/*t*HMS-CA ≥ 67% – ≥ 97%) (Ebert et al. 2022), HMS-CA **5** > 90% (unpublished data—newly investigated in the course of this study)). In plasma, glucuronide shares were noticeably lower compared to urine, which is also in agreement with the oral data. Conjugation shares observed for parent HMS after dermal (around 20%) exposure were lower than after oral exposure (*t*HMS: 53% (44–68%); in the case of *c*HMS, low concentrations hampered a reliable assessment of glucuronidation rates for the oral study). As the noxa responsible for HMS toxicity is yet unknown, the toxicological relevance of this reduced glucuronidation (and thus higher concentration of free HMS) cannot be evaluated at this time. In regard to the carboxylic acid metabolites, the mean glucuronide shares are in good agreement between the dermal (*t*HMS-CA 21%, *c*HMS-CA 10%, HMS-CA **5** 36% (17–57%)) and oral (18%, 4% (Ebert et al. 2022), and 38% (20–56%) (unpublished data)) uptake routes.

#### Comparison of metabolite ratios between the dermal and oral uptake routes

**Fig. 5:**
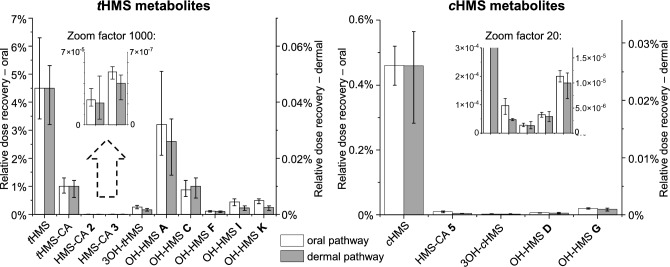
Comparison of relative dose recoveries of parent HMS and its quantitatively or semi-quantitatively analyzed specific oxidative metabolites between the oral and dermal pathways. Data are shown separately for *t*HMS (left graph) and *c*HMS (right), including their respective metabolites (OH-HMS **E** was excluded as it is most likely composed of two different co-eluting isomers—one formed from *c*HMS and the other from *t*HMS; *c*HMS-CA was excluded as it is formed from both parent HMS isomers). Data for the oral (white bars) and dermal (gray bars) pathways are plotted on two separate y-axes. For ease of comparison of the relative contributions of each metabolite to HMS elimination, axes are scaled so that the bars for parent HMS are approximately the same height for both pathways. Error bars indicate the range between minimum and maximum individual values

After the dermal application, we recovered 0.098% of the *t*HMS dose and 0.028% of the *c*HMS dose as the sum of the respective parent compound and oxidized metabolites, compared to 10.9% and 0.5% after oral dose. Hence, dermal HMS uptake was considerably lower than for the oral pathway, which is in line with studies on other compounds such as Bisphenol S (Khmiri et al. 2020), 7-hydroxycitronellal (Stoeckelhuber et al. 2018), or other UV filters (Stoeckelhuber et al. 2020; Bury et al. 2019b). In Fig. 5, the relative dose recoveries (i.e., the molar fractions of the external HMS dose excreted as the metabolites in question) of the parent HMS isomers and the respective specific oxidative metabolites are compared between the oral and dermal pathways. For this comparison, *c*HMS and *t*HMS (each including their respective metabolites) were treated separately. Two separate y-axes were used to plot the data, with scaling chosen so that the bars for parent HMS were approximately the same height for both pathways. Thus, the contribution of each metabolite to parent HMS elimination can be easily compared between the uptake routes (visually: Fig. 5, and numerically, as the ratio between the relative dose recoveries of the metabolite in question and the sum of all metabolites of the respective parent HMS isomer: Table 3). In the case of HMS-CA **5**, the F_ue_s and relative dose recoveries were obtained by quantitative analysis of pooled urine samples using a revalidated method (Ebert et al. 2023) as described in Sect. "Confirmation of HMS-CA 5 relative dose recovery". Interestingly, the investigated metabolites had comparable quantitative relevance for parent HMS elimination in both uptake routes; at most, a difference of a factor of two was observed (true for both *c*HMS and *t*HMS metabolite spectra). So, any differences in absorption, distribution, metabolism, and/or elimination (ADME) between the oral and dermal uptake routes did not have a sizeable effect on the quantitative composition of the spectrum of specific (i.e., with intact HMS core structure) metabolites (including parent HMS) of either *c*HMS or *t*HMS excreted via urine. The remaining share of the absorbed dose is probably predominantly excreted as non-specific metabolites resulting from ester cleavage into salicylic acid and homomenthol and their downstream metabolites.

**Table 3:** Relative dose recoveries of quantitatively and semi-quantitatively investigated metabolites (except for the co-eluting OH-HMS **E** isomers, and *c*HMS-CA, which is formed from both *t*HMS and *c*HMS) and parent HMS after oral dose (Ebert et al. 2022) and dermal sunscreen application (each *n* = 4)

	Relative dose recovery		Relative contribution to elimination^a^		
	Dermal (this study) (%)	Oral^b^ (Ebert et al. 2022) (%)	Dermal (%)	Oral (%)	dermal/oral ratio (F_D/O_)
*t*HMS-derived metabolites					
*t*HMS	0.045 (0.032–0.053)	4.5	46	41	1.1
*t*HMS-CA (HMS-CA **1**)	0.0098 (0.0060–0.012)	1.0	10	9.2	1.1
3OH-*t*HMS (OH-HMS **B**)	0.0017 (0.0011–0.0021)	0.26	1.7	2.4	0.7
*HMS-CA ****2***^*c*^	0.000021 (0.0000054–0.000047)	0.0024	0.021	0.022	1.0
*HMS-CA ****3***^*c*^	0.000040 (0.000024–0.000048)	0.0051	0.041	0.047	0.9
*OH-HMS ****A***	0.026 (0.014–0.034)	3.2	26	29	0.9
*OH-HMS ****C***	0.010 (0.0058–0.013)	0.88	10	8.1	1.3
*OH-HMS ****F***	0.0010 (0.00066–0.0012)	0.11	1.0	1.0	1.0
*OH-HMS ****I***	0.0023 (0.0014–0.0030)	0.45	2.3	4.1	0.6
*OH-HMS ****K***	0.0024 (0.0014–0.0030)	0.49	2.4	4.5	0.5
*Sum*	0.098	10.9			
*c*HMS-derived metabolites					
*c*HMS	0.026 (0.016–0.032)	0.46	94	92	1.0
3OH-*c*HMS (OH-HMS **H**)	0.00015 (0.000086–0.00023)	0.0028	0.54	0.56	1.0
HMS-CA **5**	0.00027 (0.00025–0.00029)^d^	0.0097^d^	1.0	1.9	0.5
*OH-HMS ****D***	0.00033 (0.00023–0.00043)	0.0065	1.2	1.3	0.9
*OH-HMS ****G***	0.0010 (0.00069–0.0012)	0.020	3.6	4.0	0.9
*Sum*	0.028	0.50			

Three of the volunteers participated in both studies, see Table S5 for individual data. Data are shown as mean values with ranges in parentheses. Semi-quantitatively analyzed metabolites are given in italics

^a^ratio between the relative dose recoveries of the metabolite in question and the sum of all metabolites of the respective parent HMS isomer (including unchanged HMS after deglucuronidation), the dermal/oral ratio indicates the differences between both uptake routes

^b^urinary excretion fractions (F_ue_) reported in Ebert et al. (2022)

^c^data for HMS-CA isomers **2** and **3** are less reliable due to overlap with *t*HMS-CA tailing, low concentrations, and a minor matrix peak co-eluting with HMS-CA **3**

^d^investigated in pool urine samples using HMS-CA **5** calibration function and revalidated method described in Ebert et al. (2023)

As mentioned in Sect. "Urinary elimination kinetics", background concentrations were observed for some HMS metabolites. However, as the metabolite concentrations in these samples were a factor of > 200 below the respective maximum concentrations after the sunscreen applications, these background concentrations had no relevant effect on the recorded kinetics and the estimated percentages of the applied dose excreted in urine. Nevertheless, background correction was performed as described in Section S-3 for the calculation of the relative dose recoveries shown in Table 3 and Fig. 5.

### Recommendations for human biomonitoring of HMS exposures

As explained in the introduction, toxicokinetic data from oral dose experiments are used in reverse dosimetry to back-calculate oral-dose-equivalent intakes from urinary biomarker data. In the previous section, we were able to demonstrate that the uptake route only moderately affected (factor of 2 differences at most) the relative contribution of each metabolite to the overall spectrum of metabolites of both *c*HMS and *t*HMS excreted in urine. We did not observe any effect of pathway-specific phase I metabolism on the relative share of systemically available *c*HMS and *t*HMS, as can be seen from the very comparable relative contribution of parent HMS elimination: 41% (40–43%) and 46% (44–51%) in the case of *t*HMS for the oral and dermal uptake routes, respectively, and 92% (91–93%) and 94% (93–94%) in the case of *c*HMS (see Table 3). Accordingly, application of the F_ue_s from the oral dosing study (Ebert et al. 2022) for reverse dosimetry (see, e.g., Lorber et al. 2017; Weschler et al. 2015) will also result in reasonably accurate estimates of exposure for the dermal route. The term ‘oral-dose-equivalent intakes’ is used to point out that exposures have been calculated using oral-route data (as described in Salthammer et al. 2018). The advantage of this approach is that it enables the direct comparison of cumulative, multi-pathway exposures with toxicological threshold values such as the TDI or RfD, generally expressed as oral daily intakes (examples for this approach can be found in Frederiksen et al. 2011; Stoeckelhuber et al. 2020; Murawski et al. 2021).

The approach described herein focuses on systemically available homosalate. It should be noted that if either salicylic acid and/or homomenthol (or their metabolites) turned out to be relevant noxae, this approach could result in an overestimation of the risk from dermal exposures. Ester hydrolysis may play a larger role for the oral uptake route than for the dermal route, as was observed for octocrylene, another UV filter (Bury et al. 2023). The current approach would then represent a conservative risk assessment.

As reported previously (Ebert et al. 2022), we try to avoid the analysis of the parent compounds in human biomonitoring as sole biomarkers of exposure due the risk of external contamination during the pre-analytical (sample collection) and the analytical phase. UV filter substances present at high concentrations in many personal care products are especially prone to this external contamination. The use of specific metabolites circumvents this problem and enables a very sensitive but still highly specific quantification of exposures. We previously identified two specific metabolites for each of the two parent isomers as rugged exposure biomarkers: the *t*HMS-derived metabolites *t*HMS-CA and 3OH-*t*HMS and the *c*HMS-derived metabolites HMS-CA **5** and 3OH-*c*HMS. Authentic analytical standards and a recently published LC–MS/MS human biomonitoring method enable their reliable quantification, sensitive enough for urine samples of the general population (Ebert et al. 2021, 2023). Based on the urinary excretion fractions and limits of quantification, the carboxylic acid metabolites *t*HMS-CA (for *t*HMS) and HMS-CA **5** (for *c*HMS) are expected to be the more sensitive biomarkers, with the OH metabolites as confirmatory biomarkers.

As shown in Table 3, the relative metabolite excretions of *t*HMS-CA and 3OH-*c*HMS are in good agreement between both uptake routes. Hence, the urinary excretion fractions (F_ue_s) determined in the oral metabolism study (Ebert et al. 2022) should be used without further adjustment. In the case of 3OH-*t*HMS and HMS-CA **5**, their relative share in excretion seems a bit smaller (factor 0.5–0.7) after dermal application than after oral uptake. The orally derived F_ue_s would, therefore, underestimate dermal uptake by a factor of up to 2. A simple adjustment can be performed, using the dermal/oral ratio (F_D/O_) for the relative contribution of these metabolites to total *t*HMS and *c*HMS elimination, respectively. For this purpose, the F_ue_ is multiplied by F_D/O_ (0.7 for 3OH-*t*HMS and 0.5 for HMS-CA **5**). The inclusion of these factors may potentially result in a slight overestimation of additionally contributing oral HMS exposure but thereby lead to a more conservative assessment for the benefit of higher consumer safety. Accordingly, oral-dose-equivalent daily intakes (DI) from spot urine samples can be calculated according to Kohn et al. (2000) using the following equation:

$${DI }_{[\mu {\text{g}}/\mathrm{kg~bw}/{\text{d}}]} = \frac{{c}_{{\text{metabolite}}}\times {CE}_{[{\text{mg}}/\mathrm{kg~bw}/{\text{day}}]}}{{F}_{{\text{ue}}}\times {F}_{{\text{D}}/{\text{O}}}\times 1000\,{\text{mg}}/{\text{g}}} \times \frac{{M}_{{\text{HMS}}}}{{M}_{{\text{metabolite}}}}$$

with c being the creatinine-adjusted metabolite concentration [µg (g creatinine)^−1^], M the molar masses of HMS (262.35 g mol^−1^) and the metabolite (HMS-CA = 292.33 g mol^−1^, OH-HMS = 278.35 g mol^−1^), CE the average creatinine excretion rates (18 and 23 mg creatinine (kg body weight)^−1^ day^−1^ for women and men, respectively (Harper et al. 1977)), F_D/O_ the dermal/oral adjustment factor as explained above, and F_ue_ the urinary excretion fraction for the metabolite (*t*HMS-CA = 1.0%, 3OH-*t*HMS 0.26%, HMS-CA **5** 0.0097%, 3OH-*c*HMS 0.0028%). If 24-h data are available, DI calculation should rather be performed using total metabolite excretion data. In such cases, the term [c_metabolite_ × CE / (1000 mg/g)] can be replaced by the total metabolite excretion (in µg (kg bw)^−1^ d^−1^; obtained by multiplying the metabolite concentration c_metabolite_ with the total 24-h urine volume and dividing by the individual’s body weight).

We previously reported the results of a pilot HBM study with volunteers from the German population (*n* = 35), performed in the spring of 2017, in which HMS biomarker concentrations were above the LOQ in precisely those three volunteers who had used sunscreen within 5 days before sample collection (Ebert et al. 2021). As a proof of principle, the spot urine sample of the volunteer (male) with the highest biomarker concentrations was chosen for exemplary DI calculation. The volunteer in question reported the use of sunscreen 4 days prior to sample collection and indeed, the metabolite concentrations are in line with those observed in the 96-h samples of the current study. The creatinine-adjusted metabolite concentrations of *t*HMS-CA, 3OH-*t*HMS, HMS-CA **5**, and 3OH-*c*HMS were 0.746, 0.159, 0.125, and 0.091 µg (g creatinine)^−1^, respectively. Using the equation above results in calculated DIs of 1.5 and 1.9 µg *t*HMS (kg bw)^−1^ d^−1^ based on *t*HMS-CA and 3OH-*t*HMS, and 53 and 70 µg *c*HMS (kg bw)^−1^ d^−1^ based on HMS-CA **5** and 3OH-*c*HMS, respectively. Taking into account the inter-individual differences observed for the relative dose recoveries and the possibility of multi-pathway exposure, these values for each parent isomer are in good agreement.

A comparison of the relative dose recoveries of parent *c*HMS and *t*HMS reveals some differences between the oral and dermal toxicokinetics (Table 3). As already discussed in the context of the oral study (Ebert et al. 2022), the ratio between the F_ue_s of *t*HMS and *c*HMS was about 10:1, which seems to be the result of a one order of magnitude lower oral bioavailability of *c*HMS (as confirmed by plasma concentration data for that study). In contrast, the relative ratio in the dermal study was about 1.7:1, indicating that *c*HMS is only about twofold less biologically available than *t*HMS for the dermal uptake route. This is reflected by the calculated oral-dose-equivalent daily intakes: the ratio between the DIs of *c*HMS and *t*HMS is in the range of 30:1 and 44:1 and thus deviates by roughly a factor of 5 from the expected ratio of 7:1 (based on the *c*HMS:*t*HMS ratio of ~ 88:12 in current sunscreen products). This indicates that the uptake of *c*HMS relative to *t*HMS is about fivefold higher for the dermal compared to the oral pathway. Conversely, the calculated oral-dose-equivalent intakes suggest that HMS exposure in the investigated sample predominantly occurred via the dermal route, in agreement with the sunscreen use reported by the volunteer in question.

### Preliminary risk assessment

To exemplify first risk assessment with these biomonitoring data, the calculated oral-dose-equivalent daily intakes will be compared with the extrapolated no-observed-adverse-effect level (NOAEL) used by the SCCS for margin of safety (MoS) calculation (20 mg (kg bw)^−1^ d^−1^) (Scientific Committee on Consumer Safety (SCCS) 2021). Using the respective higher value calculated for the oral-dose-equivalent DIs of *c*HMS and *t*HMS for a more conservative assessment results in a daily intake of 72 µg total HMS (kg bw^−1^) d^−1^, which is 278-fold below the NOAEL. However, as this calculation is only based on a single volunteer with unknown application conditions, a more detailed consideration using the experimental data described herein seemed appropriate.

Two different approaches were considered. First, the body weight-adjusted total metabolite amounts excreted over the entire sample period (96 h, background-corrected; in µg (kg bw)^−1^) were used for oral-dose-equivalent intake calculation, replacing the term [c_metabolite_ × CE / (1000 mg/g)], providing a measure of the oral-dose-equivalent intake to the total absorbed HMS dose. In a most conservative approach, it is assumed that the entire absorbed dose becomes systemically available within the first 24 h, and thus the calculated dose provides a highly conservative estimate for the oral-dose-equivalent daily intake on the day of application. Both the minimum and maximum oral-dose-equivalent DIs were calculated for each volunteer (*n* = 4) and used for MoS calculation, resulting in values ranging from 11–28 (see Table S6 for individual values).

However, it is known that the skin may serve as a reservoir (Kemppainen et al. 1991; Chu et al. 1996), making continued absorption after the first 24 h likely. Thus, in the second approach, the metabolite concentrations determined in 0–24-h pool urine samples (investigated for the determination of glucuronidation status, see S-4.2) and calculated 0–24-h urine volumes were used instead. This represents the least conservative approach as the metabolites excreted in this time frame must have been formed solely from HMS absorbed within the first 24 h. Furthermore, judging from toxicokinetics after oral dose (Ebert et al. 2022), it must be assumed that a considerable share of this systemic dose (at least 20%) will be eliminated in the form of the different HMS metabolites later than 24 h post-dose; that share of the dose will not be captured using this approach. Again, minimum and maximum oral-dose-equivalent intakes were calculated, and MoS ranged between 21 and 92 (see Table S7 for individual values).

Both approaches, therefore, show that MoS were 92 at most after a single whole-body application of 10% HMS-containing sunscreen. Given the determined half-times of HMS and its metabolites, it has to be expected that regular and repeated sunscreen use will lead to higher calculated daily intakes, and therefore lower MoS during the period of whole-body sunscreen use (e.g., during beach holidays).

## Conclusion

This study presents the first data on the diastereoselective toxicokinetics of *cis*- and *trans*-homosalate and their specific metabolites after dermal sunscreen application. Similar to oral HMS exposure, clear diastereoselectivity was observed, with the dermal bioavailability of *t*HMS being roughly twice as high as that of *c*HMS and thus confirming the necessity of the separate consideration of *c*HMS and *t*HMS in exposure assessments. The relative metabolite excretions of the investigated specific oxidative metabolites were in good agreement between the dermal and oral uptake routes. For both uptake routes, the formation and urinary excretion of specific oxidative metabolites is about tenfold more pronounced for *t*HMS than for *c*HMS. More importantly, this demonstrates the applicability of the previously determined oral-route data for the reliable assessment of dermal HMS exposures. Considering that, in practice, both the dermal and oral uptake routes will contribute to the overall HMS exposure (via, e.g., the use of lip care products with sun protection factor, the consumption of food without cutlery, and, especially in young children, hand-to-mouth activity), the equivalence in applicability of the F_ue_s for both pathways makes human biomonitoring an extraordinarily accurate tool for HMS exposure assessment.

Exemplary calculations of oral-dose-equivalent intakes demonstrated MoS between 11 and 92 after singular whole-body sunscreen application. In this context, it has to be noted that for outdoor protection, regular reapplication of sunscreen is recommended (every two hours) (NHS UK 2023; American Academy of Dermatology 2023; European Commission 2009). It remains to be seen whether the planned changes to the European Cosmetics Directive, coming into effect in 2025 and limiting HMS to facial products along with a decreased maximum concentration of 7.34%, will be sufficient to keep HMS exposure of the general population at safe levels. Accordingly, an investigation of HMS exposure in large-scale population studies or occupationally exposed individuals appears advisable.

### Supplementary Information

Below is the link to the electronic supplementary material.

Supplementary material 1 (.pdf 629 KB)

## Data Availability

Data will be made available on request.

## Abbreviations

bw: Body weight
EHS: Ethylhexyl salicylate
HMS: Homosalate
*c*HMS: *cis*-Homosalate
*t*HMS: *trans*-Homosalate
*t*HMS-CA: *trans*-Homosalate-carboxylic acid (*trans*-5-((2-hydroxybenzoyl)oxy)-3,3-dimethylcyclohexane-1-carboxylic acid)
*c*HMS-CA: *cis*-Homosalate-carboxylic acid (*cis*-5-((2-hydroxybenzoyl)oxy)-3,3-dimethylcyclohexane-1-carboxylic acid)
3OH-*t*HMS: (1*R*,3*S*)- and (1*S*,3*R*)-3-Hydroxy-3,5,5-trimethylcyclohexyl 2-hydroxybenzoate
3OH-*c*HMS: (1*R*,3*R*)- and (1*S*,3*S*)-3-Hydroxy-3,5,5-trimethylcyclohexyl 2-hydroxybenzoate
F_ue_: Urinary excretion fraction
HBM: Human biomonitoring
HMS-CA ** 5**: (1R,3S,5S)- and (1S,3R,5R)-3-((2-Hydroxybenzoyl)oxy)-1,5-dimethylcyclohexane-1-carboxylic acid
PCP: Personal care product
SCCS: Scientific Committee on Consumer Safety
UV: Ultraviolet
