# Supplementary material for: Toxicokinetics of homosalate in humans after dermal application: applicability of oral-route data for exposure assessment by human biomonitoring
Source: Arch Toxicol. 2024 Mar 14;98(5):1383–98. doi: 10.1007/s00204-024-03704-7 (PMC10965677; doi:10.1007/s00204-024-03704-7)
Supplement: Supplementary file 1 — Supplementary material 1 (.pdf 629 KB) [file 204_2024_3704_MOESM1_ESM.pdf]

Supplementary Information for the Article:

Toxicokinetics of homosalate in humans after dermal application: applicability of oral-route-data for exposure assessment by human biomonitoring

Katharina E. Ebert[a], Peter Griem[b], Tobias Weiss[a], Thomas Brüning[a], Heiko Hayen[c], Holger M. Koch[a], Daniel Bury[a]*

[a] Institute for Prevention and Occupational Medicine of the German Social Accident Insurance, Institute of Ruhr University Bochum (IPA), Bürkle-de-la-Camp-Platz 1, 44789 Bochum, Germany

[b] Symrise AG, Mühlenfeldstraße 1, 37603 Holzminden, Germany

[c] Institute of Inorganic and Analytical Chemistry, University of Münster, Corrensstrasse 48, 48149 Münster, Germany

*Corresponding author:

E-mail: daniel.bury@dguv.de

Tel.: +49 (0)30 13001 4414

Fax: +49 (0)30 13001 864414

ORCID iD: orcid.org/0000-0003-1283-3133

Table of Contents

S-1 Determination of sunscreen HMS content and HMS isomer composition.......................... 3
S-2 Additional information on quantification of analytes.. 5
S-3 Additional information on statistical analysis 7
S-4 Supplementary data on toxicokinetics 8
S-4.1 Further data on urinary elimination kinetics... 8
S-4.2 Glucuronidation of HMS metabolites and parent HMS 10
S-4.3 Relative metabolite excretion by volunteer 11
S-5 Supplementary data on preliminary risk assessment 13
References 14

S-1 Determination of sunscreen HMS content and HMS isomer composition

The HMS content and isomeric composition of the sunscreen intended for dermal application (Coppertone Sport Sunscreen Lotion SPF 50, see Table S1 for a list of the declared ingredients), containing 10% HMS according to the ingredients list, were investigated by LC-UV using an Agilent 1260 Infinity HPLC system equipped with a diode array detector (G1315B). An Accucore™ C18 column (150×3 mm, particle size 2.6 μm) was used for the chromatographic separation. Eluents were water (eluent A) and acetonitrile (eluent B), each containing 0.5% formic acid.

Table S1. Declared ingredients of the analyzed sunscreen

Sunscreen	Ingredients
Coppertone Sport Sunscreen Lotion SPF 50	Active ingredients: avobenzone 3%, homosalate 10%, octisalate 4.5%, octocrylene 8%<br>Inactive ingredients: water, aluminum starch octenylsuccinate, styrene/acrylates copolymer, glycerin, polyester-27, silica, phenoxyethanol, isododecane, arachidyl alcohol, beeswax, ethylhexylglycerin, neopentyl glycol diheptanoate, behenyl alcohol, tocopherol, acrylates/C10-30 alkyl acrylate crosspolymer, arachidyl glucoside, glyceryl stearate, PEG-100 stearate, potassium hydroxide, fragrance, disodium EDTA, sodium ascorbyl phosphate

A mixed stock solution in acetonitrile containing 100 mg L^{-1} each of *t*HMS and *c*HMS was prepared by mixing stock solutions of Neo Heliopan HMS (Symrise; 89.8% *c*HMS, 10.2% *t*HMS) and *t*HMS (TLC; 2.5% *c*HMS, 97.5% *t*HMS) (see Ebert et al. (2022) for the determination of isomer ratios) in a 10 mL volumetric flask and diluting with acetonitrile. From the mixed stock solution, calibration solutions (0, 10, 25, 50, 75, 100 mg L^{-1}) were prepared by diluting with acetonitrile.

Approximately 20 mg of the sunscreen product, weighed exactly, was diluted with acetonitrile in a 20 mL volumetric flask and vortexed for several minutes. 1 mL of the suspension was transferred into an HPLC vial and centrifuged at 1900 *g* for 10 min, then 500 µL of the supernatant was transferred into a new HPLC vial and used for analysis.

Chromatographic separation and data evaluation were performed as described previously (Ebert et al., 2022) using isocratic elution at 80% B with a flow rate of 400 μL min^{-1}. The injection volume was 5 μL and all analyses were performed in triplicate. Peak apex absorption spectra were recorded from 190 to 400 nm and absorption maxima were observed at 238 and 306 nm, which is in line with literature values (Scientific Committee on Consumer Products (SCCP) 2007) and previous observations (Ebert et al. 2021; Ebert et al. 2022). The UV absorption was measured at 234.4, 254.4 and 306.4 nm. HMS content and mean isomer ratios (based on peak areas) were calculated across all three acquisitions using the 306.4 nm signals. See Fig. S1 for exemplary chromatograms and peak apex absorption spectrum and the calibration curves. Total HMS content was 9.9%, confirming the 10% declared by the manufacturer, and the *cis:trans* isomer ratio was 87.4:12.6.

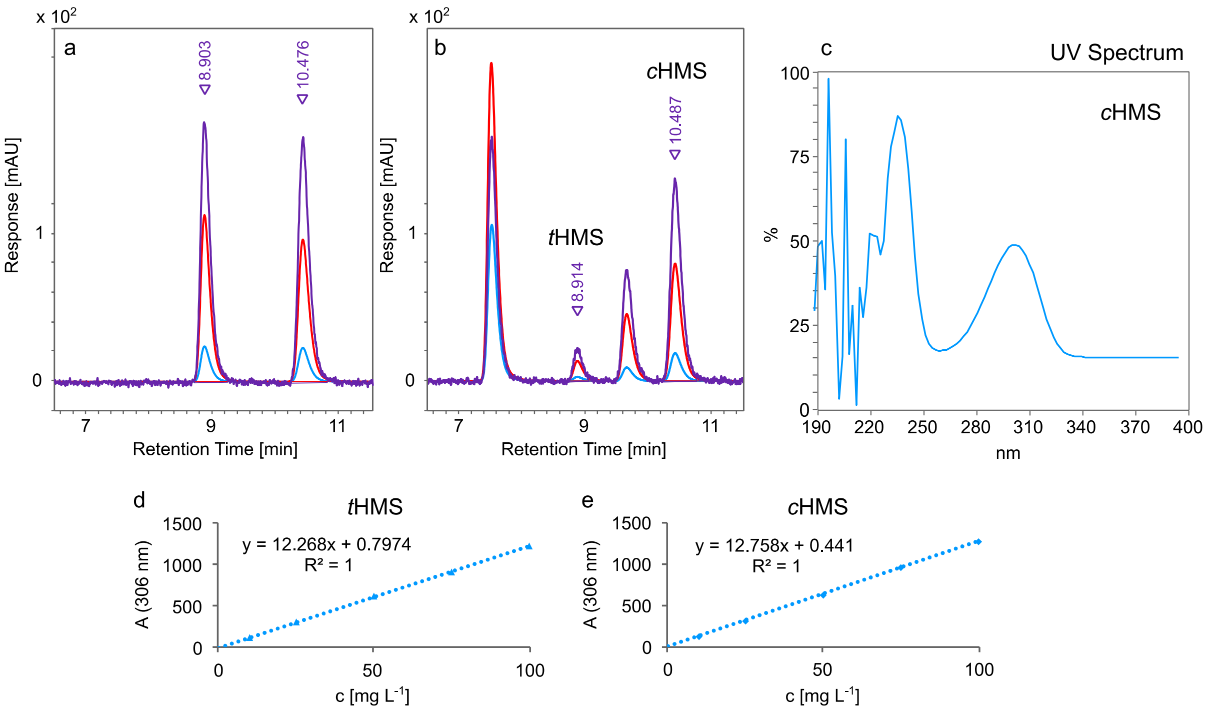

Fig. S1 Determination of HMS content via LC-UV. The subfigures show exemplary chromatograms of (a) a calibration solution containing 100 mg L^{-1} each of *t*HMS and *c*HMS and (b) a 1 g L^{-1} Coppertone Sport Sunscreen Lotion SPF 50 solution, containing 10% HMS according to ingredients list (purple: 234.4 nm, red: 306.4 nm, blue: 254.4 nm.), (c) a peak apex UV spectrum of *c*HMS, and the obtained calibration curves for (d) *t*HMS and (e) *c*HMS

S-2 Additional information on quantification of analytes

Table S2 provides an overview of the investigated metabolites and information on the analytical standards and internal standards used for quantification, as previously reported in Ebert et al. (2022).

Table S2. Summary of all analyzed metabolites including the respective calibration curves and internal standards

Analyte	Retention Time [min]	Calibration	Internal Standard
Quantitatively-analyzed metabolites			
*t*HMS-CA (HMS-CA **1**)	14.3	authentic (*t*HMS-CA)	authentic (*t*HMS-CA-d_4)
*c*HMS-CA (HMS-CA **4**)	15.3	authentic (*c*HMS-CA)	authentic (*c*HMS-CA-d_4)
3OH-*t*HMS (OH-HMS **B**)	15.5	authentic (3OH-*t*HMS)	surrogate (*c*HMS-CA-d_4)
3OH-*c*HMS (OH-HMS **H**)	19.7	authentic (3OH-*c*HMS)	surrogate (*c*HMS-CA-d_4)
*t*HMS	27.3	authentic (*t*HMS)	authentic (*t*HMS-d_4)
*c*HMS	28.5	authentic (*c*HMS)	authentic (*c*HMS-d_4)
Semi-quantitatively analyzed metabolites			
HMS-CA **2**	14.6	surrogate (*t*HMS-CA)	surrogate (*t*HMS-CA-d_4)
HMS-CA **3**	15.1	surrogate (*c*HMS-CA)	surrogate (*c*HMS-CA-d_4)
HMS-CA **5**	15.7	surrogate (*c*HMS-CA)	surrogate (*c*HMS-CA-d_4)
OH-HMS **A**	15.2	surrogate (3OH-*t*HMS)	surrogate (*c*HMS-CA-d_4)
OH-HMS **C**	15.7	surrogate (3OH-*t*HMS)	surrogate (*c*HMS-CA-d_4)
OH-HMS **D**	16.5	surrogate (3OH-*t*HMS)	surrogate (*c*HMS-CA-d_4)
OH-HMS **E**	16.9	surrogate (3OH-*t*HMS)	surrogate (*c*HMS-CA-d_4)
OH-HMS **F**	17.5	surrogate (3OH-*t*HMS)	surrogate (*c*HMS-CA-d_4)
OH-HMS **G**	18.6	surrogate (3OH-*c*HMS)	surrogate (*c*HMS-CA-d_4)
OH-HMS **I**	20.4	surrogate (3OH-*c*HMS)	surrogate (*c*HMS-CA-d_4)
OH-HMS **K**	20.9	surrogate (3OH-*c*HMS)	surrogate (*c*HMS-CA-d_4)
Qualitatively-analyzed metabolites			
arylOH-HMS **α**	21.3	none	none
arylOH-HMS **β**	22.1	none	none
arylOH-HMS **γ**	22.9	none	none
arylOH-HMS **δ**	23.6	none	none

For the confirmation of HMS-CA **5** relative metabolite excretions, the adapted human biomonitoring analytical method described in Ebert et al. (2023) was used, which includes an authentic analytical standard for HMS-CA **5** and does not detect the parent isomers *t*HMS and *c*HMS. See Ebert et al. (2023) for more detailed information. In order to show the comparability of the two methods, the metabolism samples of one volunteer were also analyzed using the adapted method. The obtained kinetics match perfectly to those obtained during the initial sample analysis (for which *c*HMS-CA was used as a surrogate calibrant for HMS-CA **5**) (see Fig. S2), apart from the offset in concentrations caused by different slopes of the calibration curves of the two analytes.

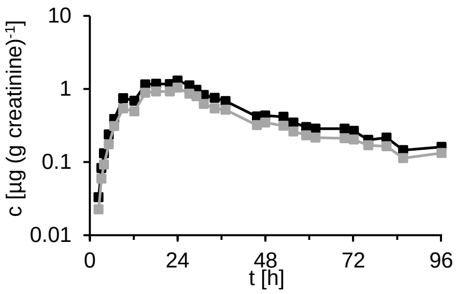

Fig. S2 Comparison of HMS-CA **5** quantification using either an authentic analytical standard or *c*HMS-CA as a surrogate calibrant. Metabolism samples of one volunteer were analyzed using the adapted human biomonitoring method as described in Ebert et al. (2023), and the obtained HMS-CA **5** excretion kinetics (black) were compared to those obtained in this study (gray)

S-3 Additional information on statistical analysis

Background subtraction

Background concentrations of several HMS metabolites were observed in three out of four volunteers. Accordingly, background correction was performed prior to calculation of relative dose recoveries as follows: Unadjusted concentrations in [µg L^{-1}] were converted to creatinine-adjusted concentrations [µg (g creatinine)$^{-1}$]. For each volunteer, the pre-dose creatinine-adjusted concentration was subtracted from all post-dose concentrations. These background-corrected concentrations were then converted back to unadjusted concentrations (c_{corr} in [µg L^{-1}]) and used for the calculation of the relative dose recovery.

Calculation of relative dose recoveries

The relative dose recovery (in %) was calculated as follows:

$$\text{Recovery} = \frac{\sum_{i=1}^{n}(c_{\text{corr}}(t_i) * V(t_i)) * M_{\text{HMS}}}{D * M_{\text{metabolite}}} \quad \text{[Equation S1]}$$

(i = i^{th} sample post-dose, n = total number of post-dose samples, t = time after sunscreen application (i.e., HMS administration), V = volume of urine sample in L, M_{HMS} = molar mass of HMS (262.35 g mol^{-1}), D = administered dose of parent isomer (*t*HMS or *c*HMS) in µg, $M_{metabolite}$ = molar mass of metabolite (278.35, 292.33, or 262.35 g mol^{-1} for OH-HMS, HMS-CA, and HMS, respectively), $c_{corr}<0$ were set as 0)

In the case of HMS-CA **5**, the dose recoveries relative to the amount of administered *c*HMS were recalculated after an authentic HMS-CA **5** analytical standard was obtained. To this end, for each volunteer 48 h and 96 h pooled urine samples were prepared from the post-dose samples of the oral and dermal metabolism studies, respectively, and analyzed using the modified and revalidated human biomonitoring method described in Ebert et al. (2023). The calculation of the relative dose recovery was performed using Equation S1 but with c_{corr} as the concentration of the respective pool urine sample (HMS-CA **5** was <LOQ in all pre-dose samples and thus no background correction was necessary) and V as the sum of the volumes of all post-dose urine samples.

S-4 Supplementary data on toxicokinetics

S-4.1 Further data on urinary elimination kinetics

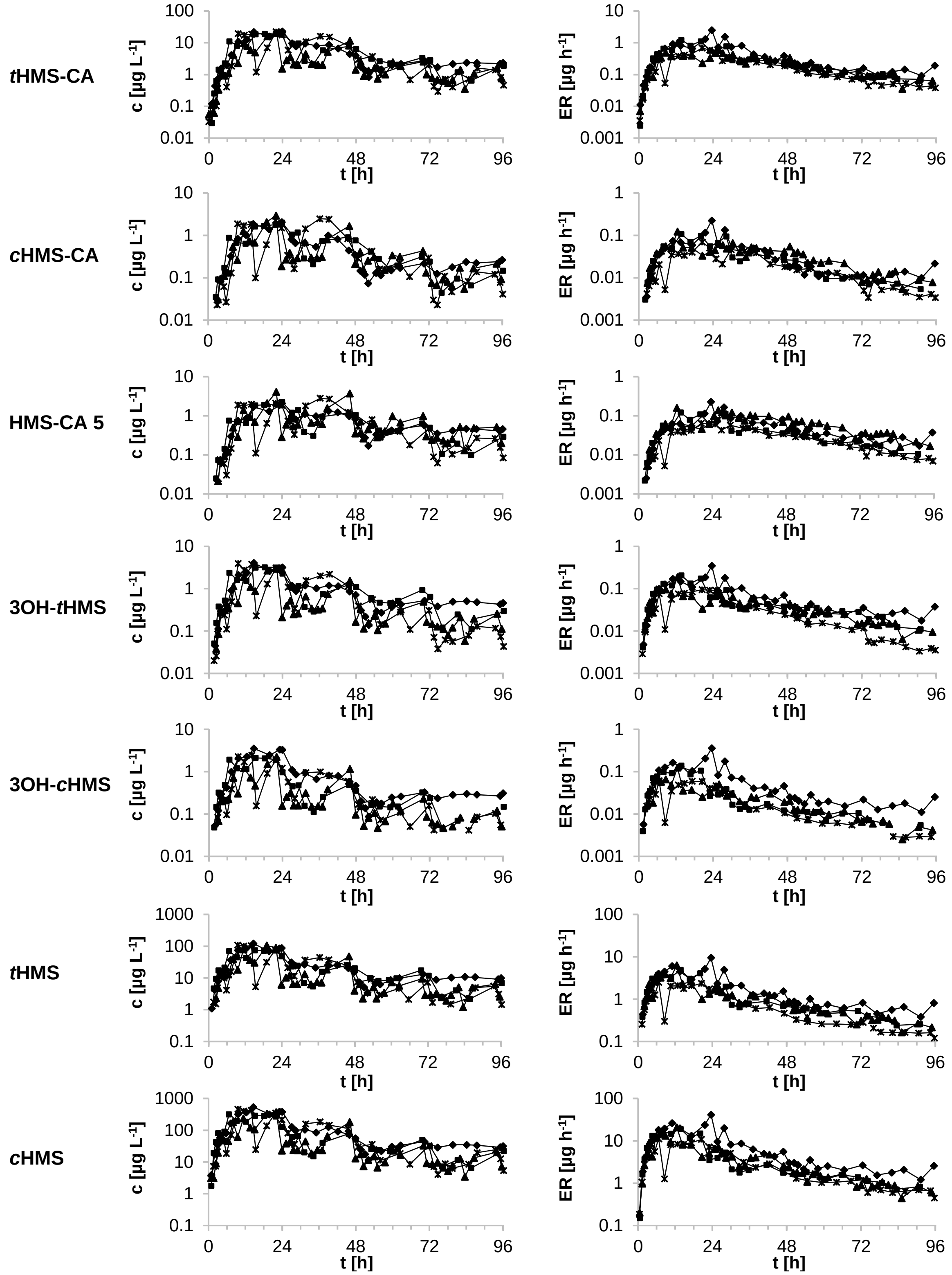

Fig. S3 Elimination kinetics of HMS and five specific metabolites in four volunteers after dermal sunscreen application (18–40 µg (kg bw)$^{-1}$). The four different data markers represent the four volunteers. The semi-logarithmic plots show the unadjusted concentration in µg L^{-1} vs. the time of sample collection (left) and the excretion rate (ER) in µg h^{-1} vs. the midpoint of each time segment (right) HMS-CA **5** was quantified using *c*HMS-CA as surrogate calibrant

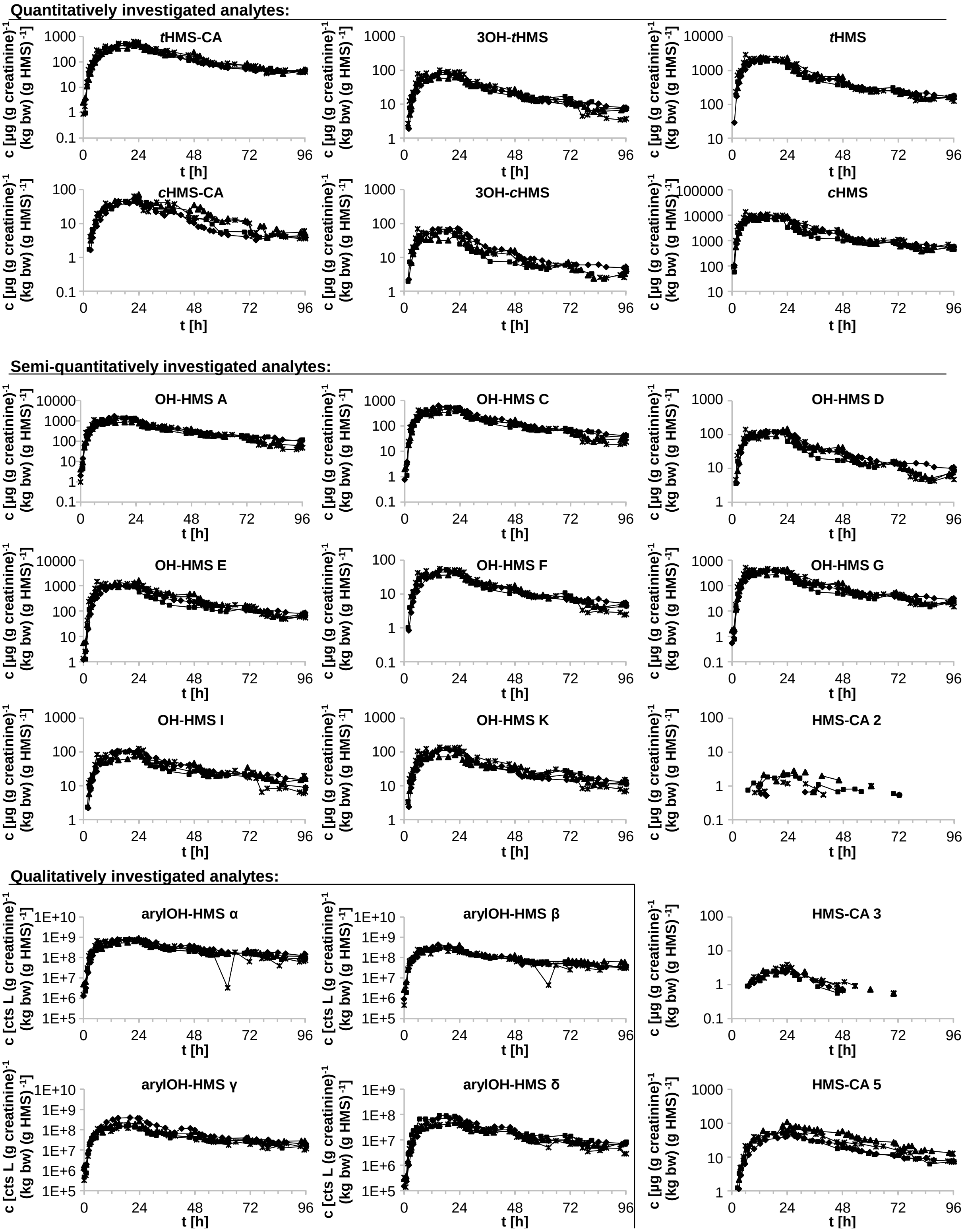

Fig. S4 Dose-normalized (i.e., divided by the body-weight adjusted dose in g HMS (kg bw)$^{-1}$) urinary elimination kinetics of HMS and its metabolites after dermal sunscreen application. Data are shown for all four volunteers after dermal application of 12.6–26.1 g sunscreen, resulting in doses of approximately 18–40 mg HMS (kg bw)$^{-1}$ (*cis:trans* isomer ratio of 87.4:12.6). The four different data markers represent the four volunteers. The semi-logarithmic plots show the creatinine-adjusted concentrations or creatinine-adjusted peak areas normalized to the applied dose of total HMS (i.e., sum of isomers), each vs. the time of sample collection

Table S3. Percentage of metabolite excreted within the first 24, 48, and 72 h post-sunscreen application (excretion after 96 h was set to 100%)

Metabolite	Excretion in %		
	**24 h**	**48 h**	**72 h**
Quantitatively analyzed metabolites			
*t*HMS	57% (55–62%)	83% (82–85%)	94% (92–94%)
*c*HMS	61% (57–70%)	85% (84–86%)	94% (94–95%)
*t*HMS-CA (HMS-CA **1**)	46% (38–54%)	80% (76–82%)	93% (93–94%)
*c*HMS-CA (HMS-CA **4**)	41% (34–48%)	78% (72–82%)	93% (92–94%)
3OH-*t*HMS (OH-HMS **B**)	49% (44–55%)	79% (75–83%)	93% (90–96%)
3OH-*c*HMS (OH-HMS **H**)	58% (49–70%)	85% (82–87%)	95% (93–97%)
Semi-quantitatively analyzed metabolites			
HMS-CA **2**[a]	41% (33–50%)	94% (81–100%)	100%
HMS-CA **3**[a]	52% (46–67%)	95% (87–100%)	100%
HMS-CA **5**	30% (22–41%)	70% (64–75%)	90% (88–91%)
OH-HMS **A**	52% (46–60%)	81% (78–82%)	94% (93–96%)
OH-HMS **C**	51% (46–60%)	81% (79–82%)	94% (93–95%)
OH-HMS **D**	55% (50–66%)	83% (81–85%)	94% (92–96%)
OH-HMS **E (E_1 + E_2)**	54% (49–66%)	83% (81–84%)	95% (92–96%)
OH-HMS **F**	48% (43–55%)	78% (74–81%)	92% (89–95%)
OH-HMS **G**	58% (52–70%)	85% (83–86%)	95% (93–96%)
OH-HMS **I**	43% (36–52%)	73% (67–77%)	90% (87–94%)
OH-HMS **K**	47% (43–53%)	76% (71–79%)	91% (89–94%)

[a] data for HMS-CA isomers **2** and **3** are less reliable due to overlap with *t*HMS-CA tailing, low concentrations, and a minor matrix peak coeluting with HMS-CA **3**

S-4.2 Glucuronidation of HMS metabolites and parent HMS

The percentages of glucuronidated species were investigated in plasma and urine by analyzing samples both with and without (i.e., equivalent volume of water added, instead of enzyme) addition of β-glucuronidase from *E. coli* K12. In addition to the quantitively investigated metabolites and parent compounds, HMS-CA **5** was investigated semi-quantitatively using the *c*HMS-CA calibration curve. The results are summarized in Table S4.

Table S4. Percentage of glucuronidated HMS and metabolites in 8 and 24 h plasma samples (n=8) and 0–24-h pooled urine samples (n=4). For urine samples, data presented as mean values and ranges if concentrations were >LOQ in all non-deconjugated samples, otherwise minimum values only

Matrix	***t*HMS-CA**	***c*HMS-CA**	**HMS-CA 5**	**3OH-*t*HMS**	**3OH-*c*HMS**	***t*HMS**	***c*HMS**
Plasma	21% (15–26%)	10% (5–23%)	36% (17–57%)[a,b]	n.a.[c]	n.a.[c]	18% (1–25%)[b]	24% (12–39%)[b]
Urine	98% (98–99%)	>93%	>93%[a]	>96%	>88%	>92%	>98%

[a] semi-quantitatively investigated using *c*HMS-CA calibration function [b] n=6/n=7 due to concentrations <LOQ after enzymatic deglucuronidation in some plasma samples, [c] could not be determined due to concentrations <LOQ in all samples, both with and without enzymatic deglucuronidation

S-4.3 Relative metabolite excretion by volunteer

Table S5. Relative dose recoveries of quantitatively and semi-quantitatively investigated metabolites (except for the coeluting OH-HMS **E** isomers, and *c*HMS-CA, which is formed from both *t*HMS and *c*HMS) and parent HMS after oral dose (Ebert et al. 2022) and dermal sunscreen application for the three volunteers who participated in both studies. Semi-quantitatively analyzed metabolites in italics

		**Relative dose recovery**		**Relative contribution to parent HMS elimination**[a]		
	**Volunteer**	**dermal (this study)**	**oral** [b] **(Ebert et al. 2022)**	**dermal**	**oral**	**ratio dermal/oral ($F_{D/O}$)**
***t*HMS-derived metabolites**						
*t*HMS	V1	0.051	6.3	44%	41%	1.1
	V2	0.053	4.8	44%	42%	1.1
	V3	0.032	3.7	51%	41%	1.2
*t*HMS-CA (HMS-CA **1**)	V1	0.012	1.3	10%	8.4%	1.2
	V2	0.011	1.1	9.2%	9.6%	1.0
	V3	0.0060	0.92	9.6%	10%	0.9
3OH-*t*HMS (OH-HMS **B**)	V1	0.0021	0.32	1.8%	2.1%	0.9
	V2	0.0019	0.26	1.6%	2.3%	0.7
	V3	0.0011	0.26	1.8%	2.9%	0.6
*HMS-CA **2***[c]	V1	0.000047	0.0018	0.041%	0.012%	3.5
	V2	0.0000054	0.0026	0.0045%	0.023%	0.2
	V3	0.000020	0.0035	0.032%	0.039%	0.8
*HMS-CA **3***[c]	V1	0.000042	0.0056	0.036%	0.036%	1.0
	V2	0.000048	0.0044	0.040%	0.038%	1.0
	V3	0.000024	0.0056	0.038%	0.062%%	0.6
*OH-HMS **A***	V1	0.032	5.1	28%	33%	0.8
	V2	0.034	3.2	28%	28%	1.0
	V3	0.014	2.3	22%	26%	0.9
*OH-HMS **C***	V1	0.012	1.2	10%	7.8%	1.3
	V2	0.013	0.91	11%	7.9%	1.4
	V3	0.0058	0.75	9.3%	8.4%	1.1
*OH-HMS **F***	V1	0.0012	0.13	1.0%	0.84%	1.2
	V2	0.0012	0.13	1.0%	1.1%	0.9
	V3	0.00066	0.12	1.1%	1.3%	0.8
*OH-HMS **I***	V1	0.0024	0.55	2.1%	3.6%	0.6
	V2	0.0030	0.52	2.5%	4.5%	0.6
	V3	0.0014	0.44	2.2%	4.9%	0.5
*OH-HMS **K***	V1	0.0030	0.55	2.6%	3.6%	0.7
	V2	0.0027	0.54	2.3%	4.7%	0.5
	V3	0.0014	0.47	2.2%	5.2%	0.4

Table S5 cont. Relative dose recoveries of quantitatively and semi-quantitatively investigated metabolites (except for the coeluting OH-HMS **E** isomers, and *c*HMS-CA, which is formed from both *t*HMS and *c*HMS) and parent HMS after oral dose (Ebert et al. 2022) and dermal sunscreen application for the three volunteers who participated in both studies. Semi-quantitatively analyzed metabolites in italics

		Relative dose recovery		Relative contribution to parent HMS elimination[a]		
	**Volunteer**	**dermal (this study)**	**oral [b] (Ebert et al. 2022)**	**dermal**	**oral**	**dermal/oral ratio ($F_{D/O}$)**
***c*HMS-derived metabolites**						
*c*HMS	V1	0.026	0.42	93%	93%	1.0
	V2	0.032	0.50	94%	92%	1.0
	V3	0.016	0.52	93%	92%	1.0
3OH-*c*HMS (OH-HMS **H**)	V1	0.00016	0.0023	0.57%	0.51%	1.1
	V2	0.00023	0.0035	0.67%	0.65%	1.0
	V3	0.000086	0.0028	0.50%	0.50%	1.0
HMS-CA **5**	V1	0.00027 [d]	0.0066 [d]	0.96%	1.5%	0.7
	V2	0.00025 [d]	0.010 [d]	0.73%	1.8%	0.4
	V3	0.00029 [d]	0.012 [d]	1.7%	2.1%	0.8
*OH-HMS **D***	V1	0.00036	0.0056	1.3%	1.2%	1.0
	V2	0.00043	0.0073	1.3%	1.3%	0.9
	V3	0.00023	0.0060	1.3%	1.1%	1.2
*OH-HMS **G***	V1	0.0012	0.018	4.3%	4.0%	1.1
	V2	0.0012	0.021	3.5%	3.9%	0.9
	V3	0.00069	0.022	4.0%	3.9%	1.0

[a] ratio between the relative dose recoveries of the metabolite in question and the sum of all metabolites of the respective parent HMS isomer (including unchanged HMS after deglucuronidation), the dermal/oral ratio indicates the differences between both uptake routes; [b] urinary excretion fractions (F_{ue}) reported in Ebert et al. (2022); [c] data for HMS-CA isomers **2** and **3** are less reliable due to overlap with *t*HMS-CA tailing, low concentrations, and a minor matrix peak coeluting with HMS-CA **3**; [d] investigated in pool urine samples using HMS-CA **5** calibration function and revalidated method described in Ebert et al. (2023);

S-5 Supplementary data on preliminary risk assessment

Table S6. Volunteer-specific parameters and calculation of oral-dose-equivalent intakes using a most conservative approach (i.e., assuming the entire absorbed dose becomes systemically available within the first 24 h)

		amount of metabolite excreted over 96 h [µg]				oral-dose-equivalent intake [µg parent isomer/kg bw/d]				oral-dose-equivalent intake [µg *c*+*t*HMS/kg bw/d]		MoS	
Volunteer	bw [kg]	*t*HMS-CA	3OH-*t*HMS	HMS-CA **5**	3OH-*c*HMS	*t*HMS-CA	3OH-*t*HMS	HMS-CA **5**	3OH-*c*HMS	min	max	min	max
V1	101	30.5	5.11	3.70	2.73	27.1	26.2	677.8	909.2	704	936	21	28
V2	95	38.0	6.12	4.57	5.11	35.9	33.4	890.2	1812.2	924	1848	11	22
V3	65	22.1	3.69	5.58	2.09	30.5	29.4	1587.6	1083.8	1113	1618	12	18
V5	68	18.2	2.89	2.59	1.65	24.0	22.0	704.8	816.6	727	841	24	28

Table S7. Volunteer-specific parameters and calculation of oral-dose-equivalent intakes using the least conservative approach (i.e., only considering the fraction of the dose that becomes systemically available within the first 24 h)

			metabolite concentration in 0–24 h pool urine [µg L^{-1}]				oral-dose-equivalent intake [µg parent isomer/kg bw/d]				oral-dose-equivalent intake [µg *c*+*t*HMS/kg bw/d]		MoS	
Volunteer	V [mL][a]	bw [kg]	*t*HMS-CA	3OH-*t*HMS	HMS-CA **5**	3OH-*c*HMS	*t*HMS-CA	3OH-*t*HMS	HMS-CA **5**	3OH-*c*HMS	min	max	min	max
V1	1635	101	9.92	1.76	0.85	1.18	14.4	14.8	254.6	643.0	269	658	30	74
V2	1764	95	9.15	1.53	0.77	1.47	15.2	14.7	263.9	918.8	279	934	21	72
V3	2858	65	2.88	0.56	0.42	0.35	11.4	12.8	341.7	516.5	353	529	38	57
V5	2297	68	3.67	0.63	0.33	0.45	11.1	11.0	205.6	511.7	217	523	38	92

[a] Total 0–24 h post-dose urine volume

References

Ebert KE, Belov VN, John M, Weiss T, Brüning T, Hayen H, Koch HM, Bury D (2023) Identification, organic synthesis, and sensitive analysis of a cis-homosalate-specific exposure biomarker. Chem. Res. Toxicol. (recently accepted)

Ebert KE, Belov VN, Weiss T, Brüning T, Hayen H, Koch HM, Bury D (2021) Determination of urinary metabolites of the UV filter homosalate by online-SPE-LC-MS/MS. Anal. Chim. Acta 1176:338754. doi: 10.1016/j.aca.2021.338754

Ebert KE, Griem P, Weiss T, Brüning T, Hayen H, Koch HM, Bury D (2022) Diastereoselective metabolism of homomenthyl salicylate (homosalate): Identification of relevant human exposure biomarkers. Environ. Int. 170:107637. doi: 10.1016/j.envint.2022.107637

Scientific Committee on Consumer Products (SCCP) (2007) Opinion of the SCCP on homosalate, COLIPA n° S12. https://ec.europa.eu/health/ph_risk/committees/04_sccp/docs/sccp_o_097.pdf. Accessed October 2022
